# Continuous glucose monitoring in non-insulin-dependent type-2 diabetes – rationale for CGM-NIDDM study

**DOI:** 10.1186/s13098-025-01976-2

**Published:** 2025-10-16

**Authors:** Carolin Rädel, Andreas Wienke, Rainer U Pliquett

**Affiliations:** 1 Martin-Luther-University Halle-Wittenberg, Ernst-Grube-Straße 40, Halle (Saale), 06120 Germany; 2 MVZ Gemeinschaftslabor Outpatient Clinic, Uhlandstr. 53, Cottbus, 03050 Germany; 3 Department of Nephrology, Diabetology, and Nephrologic Geriatry, Elblandklinik Riesa, Weinbergstr.8, Riesa, 01589 Germany; 4 Institute of Medical Epidemiology, Biometry and Informatics, Martin-Luther-University Halle-Wittenberg, Ernst-Grube-Straße 40, Halle (Saale), 06120 Germany

**Keywords:** Non-insulin-dependent type-2 diabetes mellitus, Prediabetes, Haemoglobin A1c, Continuous glucose monitoring, Nutrition counseling

## Abstract

Type-2 diabetes is treated by general practitioners and diabetologists in a stepwise manner with an emphasis on lifestyle modifications. Glucose monitoring is mandatory to gauge the effect of lifestyle modifications or medical therapies in order to identify the need for therapy escalation, if treatment goals are not met. To date, three-monthly hemoglobin A1c measurements have been conducted for this purpose in the majority of non-insulin-dependent type-2 diabetes patients in Germany. Here, we review the role of continuous glucose monitoring in combination with nutrition counseling to offer a biofeedback. In addition, we present the rationale for a hypothesis-generating study that randomizes continuous glucose monitoring or no continuous glucose monitoring to the standard of nutrition counseling in individuals with prediabetes or non-insulin-dependent type-2 diabetes mellitus.

## Background

We present a review and hypothesis for a proposed randomized clinical study on the benefit of nutrition counseling in combination with a continuous glucose monitoring in non-insulin-dependenttype-2 diabetics and prediabetics. As a goal, we aim to identify a possible optimization of therapy compared to the standard of care with regard to hemoglobin A1c. This review will be of great interest to both diabetologists and general practitioners, as the proposed study will be conducted in a general practice setting. As for continuous glucose monitoring, it has been used primarily in insulin-treated diabetes patients meaning that its full potential has not been exploited so far. The aim of this review is to work on the research question of whether or not there is a possible benefit from nutrition counseling in terms of less hyperglycemia as documented by serial haemoglobin A1c values, if a one-time continuous glucose monitoring is added. The role of biofeedback is discussed.

## Introduction

Diabetes is a major noncommunicable disease that is spreading worldwide. In 2021, the prevalence of type-1 or type-2 diabetes worldwide was 537 million adults aged 20–79, and type-2 diabetes accounted for more than 90%. By 2048, the number of persons with diabetes who are 20–79 years old will rise to 783 million [1].

Being at an elevated risk for cardiometabolic complications or for diabetes, patients with prediabetes represent an important target to monitor as well. As there is no uniform definition of prediabetes, the International Diabetes Federation (IDF) has published data on the prevalence of impaired fasting glucose (IFG) and impaired glucose tolerance (IGT) [1]. The prevalence of prediabetes is comparable to the one of overt diabetes. In 2021, there were 541 million adults with IGT and 319 million with IFG worldwide [1]. Thus, the number of persons with a need for glucose monitoring may double, if persons with type-2 diabetes and prediabetes are considered, instead of persons with a known type-2 diabetes alone.

In Germany, according to national surveys or health insurance data [2], 10% of the population is diagnosed with diabetes mellitus. In accordance with the worldwide projections, an increasing prevalence of type 2 diabetes is expected as well. The projections range between 10.7 million and 12.3 million persons with type-2 diabetes for a total population of about 85 million in Germany in 2040 [3].

According to International Guidelines [4, 5], type-2-diabetes therapy is being allocated in a stepwise manner, but in an individualized way. Lifestyle modifications, including nutrition counseling and recommendations to regularly perform exercise, represent basic therapies. If basic therapy does not suffice, oral diabetes medications are considered. As a next step, subcutaneous glucagon-like-peptide receptor agonist or a combination of glucagon-like-peptide receptor agonist and gastric inhibitory polypeptide agonist treatments are begun, if oral diabetes medications and lifestyle modifications do not suffice or are contraindicated. Insulin represents the last choice of diabetes medications, if all of aforementioned therapies do not yield the desired treatment effect. Importantly, the basic therapy of type-2 diabetes is to be maintained, irrespective of medical therapy. As a caveat, until now, a biofeedback on nutrition counseling as part of basic therapy is not available. Therapy control of basic therapy, i.e. of lifestyle including an increased physical activity and a life-long diabetes-conform diet, solely relies on haemoglobin A1c (HbA1c) measurements being performed every 3 months. Moreover, if oral diabetes medications are added to meet treatment goals, the majority of individuals with type-2 diabetes does not change the above-mentioned therapy monitoring, even though an occasional self-monitoring of blood glucose (SMBG) is recommended there. In the following section, we review the literature on the current shortcomings of patient care in non-insulin-dependent type 2 diabetes mellitus (NIDDM). Finally, we provide the rationale for a hypothesis-generating clinical study to improve biofeedback in persons with NIDDM or prediabetes, thus alleviating the issues associated with therapy escalation towards insulin.

## Method

A literature search using Google Scholar and PubMed was performed using the following key words combinations: non-insulin-dependent Type-2 diabetes mellitus AND continuous glucose monitoring (CGM), prediabetes AND continuous glucose monitoring. All randomized clinical trials (RCT) were considered for this review. Case reports and non-randomized studies were excluded from this review.

## Review of the literature

As Table 1 shows, 4 RCT on the use of CGM in NIDDM were identified, none on the use of CGM in prediabetes. Furthermore, no study has been published on the use of CGM-enhanced nutrition counseling nor on a follow-up of more than 6 months. From the literature, CGM was shown to improve outcome in NIDDM on SMBG. A meta-analysis on 407 NIDDM individuals from 4 RCT on real-time CGM and 2 RCT on intermittent-scanning RCT showed a better outcome in terms of HbA1c.reduction by 0.3% [6].

**Table 1:** Comparison of randomized clinical trials (RCT) on the use of continuous glucose monitoring (CGM) versus standard of care (haemoglobinA1c, HbA1c; selfmonitoring of blood glucose, SMBG) in non-insulin-dependent type-2 diabetes mellitus (NIDDM)

RCT	Comparison	‍Number of Patients studied	‍Outcome parameter	‍Outcome	‍Reference
NIDDM patients	CGM versus SMBG	‍93	‍HbA1c after 6 months	‍−0.5% versus − 0.2% HbA1c reduction (*p* < 0.05)	‍[18]
NIDDM patients	CGM versus SMBG	‍223	‍HbA1c after 6 months	‍−1% versus − 0.5% HbA1c reduction (*p* < 0.01)	‍[23]
NIDDM patients	CGM + telemonitoring versus SMBG + telemonitoring	‍86	‍HbA1c after 3 months	‍−0.7% versus − 0.3% HbA1c reduction (*p* < 0.01)	‍[24]
NIDDM patients	3x CGM at 0, 4, 8 weeks versus SMBG	‍68	‍HbA1c after 3 months	‍−0.5% versus − 0.2% HbA1c reduction (*p* = 0.12)	[25]

### HbA1c as a therapy control of type-2 diabetes

HbA1c measurements do not offer any biofeedback on the quality of the lifestyle modifications in type-2-diabetes patients and do not differentiate between stable or unstable plasma glucose concentrations over time [7]. Ultimately, if diabetes therapy is being escalated towards insulin, insulin-mediated weight gain and hypoglycemic episodes may occur, regardless of whether three-monthly HbA1c targets were met or not. Lifestyle modifications represent the first choice of therapy adjustment in type-2 diabetes, as lifestyle modifications do have the potential to postpone a medical-therapy escalation and its possible side effects.

### Case report: Type-2 diabetes with dietary incompliance

As anecdotal evidence a 65-year-old type-2 diabetes patient on oral diabetes medications from our own practice showed the following HbA1c values: in august 2020–6.0%, in March 2021 − 7.6%, in June 2021–6.5%, and in October 2021–7.8%. So far, therapy remained unchanged. Surprisingly, in January 2022, HbA1c rose to 10.7% due to incompliance over the holiday and year-end season. In three ensuing quarters, after nutritional counseling, HbA1c values stabilized by 7.2%, 7.5% and 7.1%.

What is the lesson learned? When relying on three-monthly HbA1c monitoring without biofeedback, it becomes clear that the HbA1c fluctuates within a range from 6.0% to 10.7% due to dietary incompliance. Thus, the patient advice should include nutrition counseling. On the other hand, prevention of microvascular complications is mandatory. Therefore, a therapy escalation rather than relying on lifestyle modifications and on oral antidiabetic medications could be the preferred choice as well. As for this case, the treating physician opted for nutrition counseling alone. Only six months earlier, the patient was within or below target range of HbA1c, i.e. 6.5% to 7.5%. Thus, in the present case, nutrition counseling proved effective. However, the issue remains that glucose spikes cannot be detected timely when relying on HbA1c alone. An analysis of possible causes for HbA1c deterioration can only be performed weeks later, when the hyperglycemic events have already gone undetected.

### Continuous glucose monitoring for biofeedback

Hypothetically, the provision of biofeedback on lifestyle modifications via CGM would increase the awareness for life-style modifications per se in persons with type 2 diabetes. In addition, biofeedback would help avoid unnecessary therapy escalations in cases of dietary incompliance.

By the means of CGM for biofeedback, the patient may learn about the nutritional effects of sugar-sweetened beverages and other convenience food with a high sugar content. When compared to nutrition counseling without one-time CGM, a biofeedback mechanism offered by CGM may engage the patient in a learning process. At the best scenario, the patient may get involved into diabetes care on a long-term basis.

### Gaps in the current patient care of type-2 diabetes

In German hospitals, approximately 3 million type-2-diabetes patients are admitted every year, irrespective of the actual cause of admission [8]. Thereof, approximately 6000 diabetics were hospitalized annually in a large community-based hospital (Medical University Lausitz - Carl-Thiem, Cottbus/Germany). This hospital was certified by the German Diabetes Association in 2002 for being suitable to diabetes patients [9]. Based on our own unpublished data from a 4-year period (2018–2022), diabetes therapy had to be escalated in a considerable proportion of roughly 10% in newly admitted, non-insulin-dependent type-2 diabetics due to hyperglycemic episodes on admission or during the hospital stay.

**Fig. 1:**
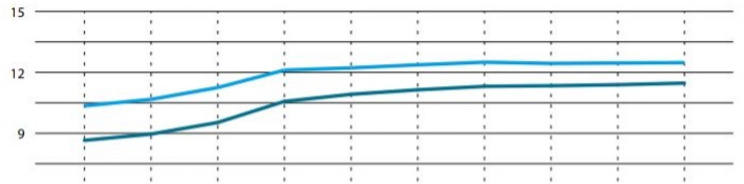
Percentage (y axis) of individuals with diabetes mellitus (light blue: all types of diabetes, dark blue: type-2 diabetes) in the German federal state of Brandenburg over time from 2007 to 2016, modified from figure 2-2. from (10). X axis: each line represents one year.

In addition, as Fig. 1 demonstrates, diabetes prevalence dramatically increased during the last decade in the respective German federal state [10]. That is, the current therapy control based on three-monthly HbA1c may not be sufficient to detect clinically relevant hyperglycemia in a timely manner. These numbers further show that little or no emphasis is laid upon basic therapy of type-2 diabetes, which is deemed to be the most important step in patient care of type-2 diabetes.

A recent study on the quality of care of diabetes patients in Germany revealed deficits in patient care and a need for improvement. Patients themselves considered the quality of care to be mediocre averaging at 2,43 in an assessment from 1 to 5 of the Assessment of Chronic Illness Care – DAWN short form. The explanation may be country-specific with shortcomings in patient care on the provider side: health-care provider gets reimbursed rather on laboratory findings (HbA1c) than by the treatment results (quality of life etc.). To this point, an improvement in medical care with regard to a stronger orientation towards the patient needs is warranted [11]. The use of CGM with the advantages of self-control and visualized aims such as time in range, time in hypoglycemia, and time in hyperglycemia can be part of this improvement process.

### Prevention of complications through optimal blood glucose control using continuous glucose monitoring

Hyperglycemic episodes may harm endothelial cells, mesangial cells in the renal glomeruli, neurons, and Schwann cells in peripheral nerves, which are unable to regulate glucose uptake. Furthermore, oxidative stress incurred by an increased glycemic variability may accelerate atherosclerosis [12]. Because of the pathophysiological impact of fluctuating blood glucose and glucose spikes, the reliance on HbA1c target values may not be sufficient to prevent diabetes-related microvascular and macrovascular complications, as complications may occur even in patients in whom the HbA1c is within target range [13]. Besides reaching a target HbA1c, CGM-based parameters of euglycemia including the “time in range” parameter may be advantageous for the quality control of diabetes therapy [14] and reduce oxidative stress via less glycemic variability [15]. Hypothetically, using CGM as a means to optimize nutrition counseling via biofeedback, glucose spikes and complications may be better addressed. Thus, an effective, CGM-enhanced nutrition counseling early on in prediabetes or in NIDDM needs to be assessed in clinical studies. If results of such a study are positive, CGM-enhanced nutrition counseling may support the standard of care which relies on regular HbA1c measurements or SMBG to control for treatment efficacy and to lower the incidence of micro- and macrovascular diabetes complications in NIDDM. So far, the use of CGM in insulin-treated type-2 diabetics, CGM was shown to improve HbA1c [16]. An explanation for this observation is referred to a more precise insulin therapy. However, a more precise dietary control and a more motivated use of exercise as basic therapies for type-2 diabetes represent additional explanations. In young type-2 diabetes patients on insulin for at least 3 months, a single-use CGM alone did not improve mid-term or long-term glycemic endpoints [17]. Thus, as for every therapeutical measure, compliance needs to be assessed, before a judgment on treatment effect is possible.

As for CGM in NIDDM with a mid-term follow-up of 6 months, Wada, E et al. [18], showed that CGM use associates with an improved glycemia after 6 months in non-insulin-treated type-2 diabetics. Here, NIDDM patients monitored by CGM were compared to a control group using SMBG. The most relevant result was seen in the HbA1c difference between groups. After 12 weeks, HbA1c was reduced in both cohorts. Intriguingly, there was a sustained effect in the CGM group beyond 12 weeks. Whether or not this promising CGM effect carries on beyond 24 weeks, is an open research question. It is also unclear, whether an elevated starting HbA1c carries the biggest treatment effect, as the included patients in the study by Wada et al. [18] were limited to patients with a maximal HbA1c of 8.5%. As accumulating evidence, an Advanced Technologies & Treatments for Diabetes (ATTD) consensus statement on “Use of Continuous Glucose Monitoring” concluded that CGM may yield a benefit in type-2-diabetes therapy but calls for more data, especially for NIDDM [19]. Carlson et al. showed a benefit for real-time CGM with regard to glucose variability in type 2 diabetic patients on oral diabetes medications [20]. As for digital literacy when using CGM, the patient contributes to a participative relationship with a higher level of health literacy and empowerment, especially for the elderly [21]. Therapy decisions promoted by all major diabetes associations depend on life expectancy, comorbidities, hypoglycemia risk, social settings and patient requests [22]. Thus, the use of CGM may further enhance patient autonomy because the patient learns much about their own body and how the body reacts to stress, sport or to different foods via biofeedback.

### Rationale for and objectives of planned randomized clinical study

We hypothesize here that CGM enables patients with noninsulin-dependent type-2 diabetes or prediabetes to better understand the value of diet and exercise for glucose control by direct feedback. As an assumption, the patient will learn, how nutrition, stress, exercise affect the glucose curve. Hypothetically, a one-time use of CGM over 2 weeks will enable better mid-term glucose control over the ensuing year in terms of HbA1c values. To study this in a randomized fashion, a study group of individuals with prediabetes or noninsulin-dependent type-2 diabetes using CGM plus standard of care and another group of individuals with prediabetes or noninsulin-dependent type-2 diabetes controlled by standard of care treatment without CGM are proposed. The hypothesis of HbA1c improvement assumes that the active comparator group receives better feedback on glucose metabolism via CGM in conjunction with nutrition counseling. Consequently, if this hypothesis holds true, CGM plus nutrition counseling could improve the basic therapy for type-2 diabetes. In addition, treating family physicians will have the possibility to set new therapy goals early on, before HbA1c increases.

To test this hypothesis, we plan to conduct a prospective, monocentric, two-arm, randomized, open-label, 12-month treatment optimization study. Cohorts are scheduled with 1:1 randomization. For the sample size calculation, the expected 12-months effect of HbA1c was based on study results of a 6 month effect of a one-time CGM on HbA1c in a similar setting [18]. Specifically, according to that study, the mean HbA1c (± standard deviation after 6 months was 7.37%±0.25 in the CGM group versus 7.67%±0.27 in the CGM-free group. The probability of a type-1 error was set to 5% and the power to 80%. This results in a sample size of 14 patients per group. Taking into account 20% drop-out, 18 patients per group will be included. Again, the assumption being made here is that the observed effect on HbA1c will last up to 12 months.

The patients are recruited by an internal data base available for the disease management program for type-2 diabetes for every German practice.

### Protocol of the proposed study on CGM in individuals with non-insulin-dependent type-2 diabetes mellitus and prediabetes

**Fig. 2:**
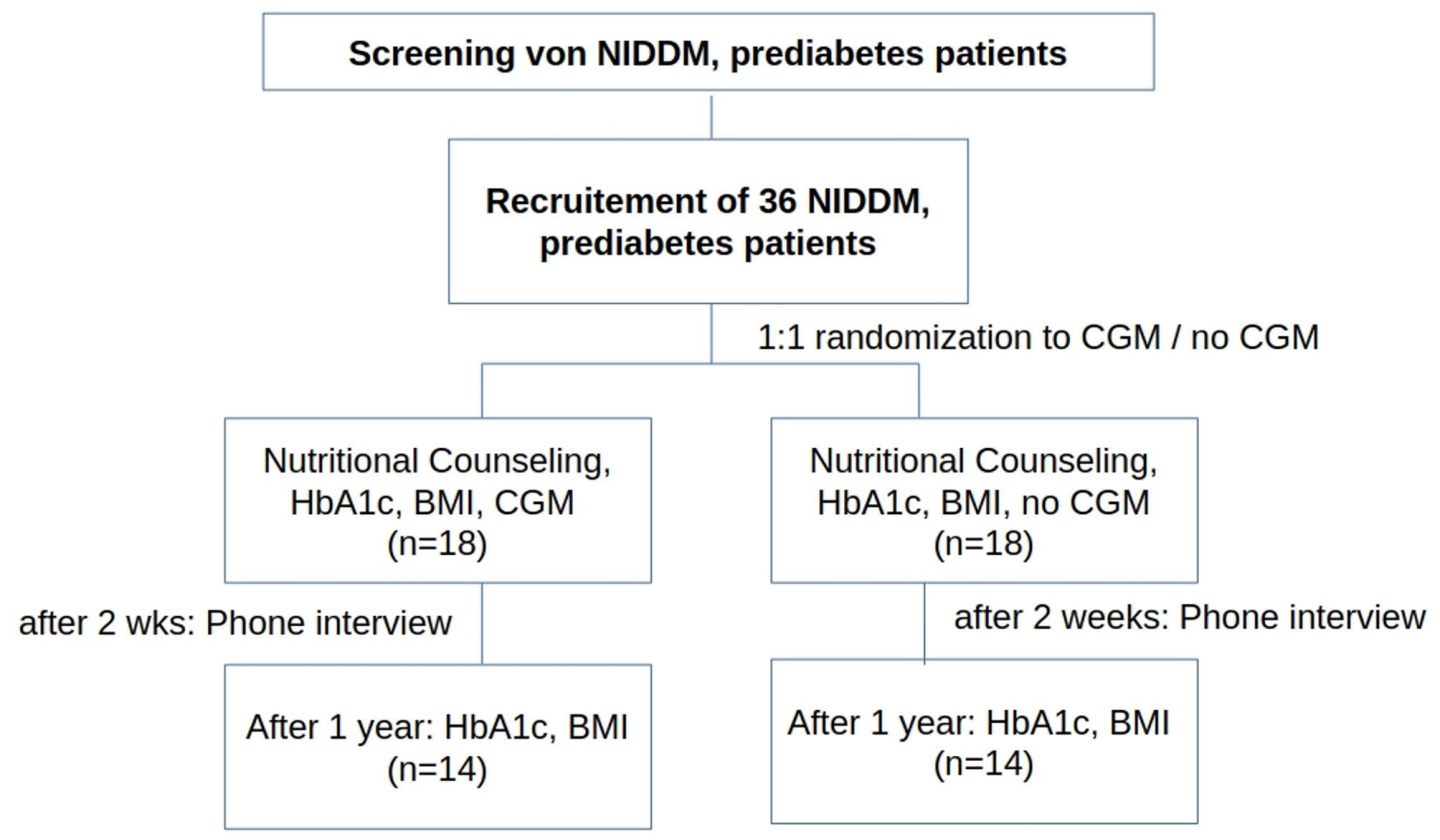
Study flow of the planned CGM-NIDDM study. A drop-out of 20% is assumed. BMI = Body-mass index, CGM = continuous glucose monitoring, NIDDM = non-insulin-dependent diabetes mellitus Type 2

Figure 2 displays the study flow of the proposed CGM-NIDDM study. There, every trial participant is provided with a thirty-minute nutrition counseling at the first appointment. Great care is given to provide a comparable quality of nutrition counseling. After that counseling, a study nurse randomizes blind all trial participants in one of the two cohorts with prepared randomization envelopes. The CGM group will get a second appointment for installation of the CGM sensor and to provide guidance for the fourteen days of CGM. After fourteen days, a short telephone feedback interview is conducted with all study participants. All of them get a blood sampling as part of the disease management program within three months at their regular visits. The periodic laboratory results serve as database. At the 12-months follow-up exam, HbA1c and changes in medication will be recorded. As primary endpoint, the 12-month HbA1c is considered. As secondary endpoints, the 3-months-, 6-months- and 9-months HbA1c are compared between both groups. Body weight (body-mass index) after 12 months versus baseline is compared.

The aim of this hypothesis-generating study is to provide the basis for an endpoint-driven randomized clinical trial with the goal of optimizing therapy in prediabetes and in noninsulin-dependent type-2 diabetes. As for prediabetes, a subgroup analysis for patients with impaired fasting glucose alone, with impaired glucose tolerance alone or with both features will be performed. Again, the patient-side optimization of basic therapy in terms of nutrition by biofeedback is the overall study goal. The general-practitioner-based analysis of the one-time CGM provided by the patient may reinforce the biofeedback to the patient.

## Discussion

In NIDDM and in prediabetes, blood glucose spikes are difficult to control by determination of HbA1c plasma levels every three months. In general, CGM may close this gap in patient care, if the results of the proposed study hold true. There, we hypothesize that dietary counseling accompanied by one-time CGM for biofeedback may improves HbA1c after a 12-months period when compared to nutrition counseling alone. The reasons, why this hypothesis is put forward, are plentiful. Altogether, they circulate around biofeedback. In addition, the use of a control group with a blinded CGM may further improve the study quality as it controls for any placebo effect inherent to a new technology on the patient side.

In addition, for the proposed study, patient recruitment should avoid any “healthier patient” bias and consider socioeconomic factors. In addition, as for physical activity, a patient diary and/or activity tracker should be applied. CGM may improve quality of care via an increased patient autonomy. In addition, as for health-care providers, CGM results may be readily available both in practice and remotely.

In line with this perspective, possible benefits of a combined use of CGM and nutrition counseling in NIDDM and in prediabetes need to be confirmed in a large, real-life study. The proposed, relatively small, controlled study represents a hypothesis-generating study and prerequisite for such a larger trial. Clearly, any future, larger study on the use of CGM in prediabetes and in NIDDM may need check additional endpoints including the roles of CGM on the exercise level achieved. In general, a new role of CGM to assist nutrition counseling may evolve.

## Data Availability

The datasets used for this review are available from the corresponding author on reasonable request.

## Abbreviations

BMI: Body-mass index
CGM: Continuous glucose monitoring
HbA1c: Haemoglobin A1c (A1c)
IDF: International diabetes federation
IFG: Impaired fasting glucose
IGT: Impaired glucose tolerance
NIDDM: Non-insulin-dependent diabetes mellitus
RCT: Randomized clinical trials
SMBG: Self-monitoring of blood glucose
